# Nonlinear functional network connectivity in resting functional magnetic resonance imaging data

**DOI:** 10.1002/hbm.25972

**Published:** 2022-06-28

**Authors:** Sara M. Motlaghian, Aysenil Belger, Juan R. Bustillo, Judith M. Ford, Armin Iraji, Kelvin Lim, Daniel H. Mathalon, Bryon A. Mueller, Daniel O'Leary, Godfrey Pearlson, Steven G. Potkin, Adrian Preda, Theo G. M. van Erp, Vince D. Calhoun

**Affiliations:** ^1^ Tri‐institutional Center for Translational Research in Neuroimaging and Data Science (TReNDS) Georgia State, Georgia Tech, Emory Atlanta Georgia USA; ^2^ Department of Psychiatry University of North Carolina Chapel Hill North Carolina USA; ^3^ Department of Psychiatry University of New Mexico Albuquerque New Mexico USA; ^4^ Department of Psychiatry University of California San Francisco San Francisco California USA; ^5^ San Francisco VA Medical Center San Francisco California USA; ^6^ Department of Psychiatry University of Minnesota Minneapolis Minnesota USA; ^7^ Department of Psychiatry University of Iowa Iowa City Iowa USA; ^8^ Department of Psychiatry and Neurobiology Yale School of Medicine New Haven Connecticut USA; ^9^ Department of Psychiatry and Human Behavior University of California Irvine Irvine California USA

**Keywords:** functional network connectivity, mutual information, nonlinear functional network connectivity, time courses

## Abstract

In this work, we focus on explicitly nonlinear relationships in functional networks. We introduce a technique using normalized mutual information (NMI) that calculates the nonlinear relationship between different brain regions. We demonstrate our proposed approach using simulated data and then apply it to a dataset previously studied by Damaraju et al. This resting‐state fMRI data included 151 schizophrenia patients and 163 age‐ and gender‐matched healthy controls. We first decomposed these data using group independent component analysis (ICA) and yielded 47 functionally relevant intrinsic connectivity networks. Our analysis showed a modularized nonlinear relationship among brain functional networks that was particularly noticeable in the sensory and visual cortex. Interestingly, the modularity appears both meaningful and distinct from that revealed by the linear approach. Group analysis identified significant differences in explicitly nonlinear functional network connectivity (FNC) between schizophrenia patients and healthy controls, particularly in the visual cortex, with controls showing more nonlinearity (i.e., higher normalized mutual information between time courses with linear relationships removed) in most cases. Certain domains, including subcortical and auditory, showed relatively less nonlinear FNC (i.e., lower normalized mutual information), whereas links between the visual and other domains showed evidence of substantial nonlinear and modular properties. Overall, these results suggest that quantifying nonlinear dependencies of functional connectivity may provide a complementary and potentially important tool for studying brain function by exposing relevant variation that is typically ignored. Beyond this, we propose a method that captures both linear and nonlinear effects in a “boosted” approach. This method increases the sensitivity to group differences compared to the standard linear approach, at the cost of being unable to separate linear and nonlinear effects.

## Graphical abstract

In this work, we focus on explicitly nonlinear relationships in functional networks. We introduce a normalized mutual information (NMI) technique that calculates the nonlinear relationship between different brain regions. Also, we propose a method that captures both linear and nonlinear effects in a “boosted” approach. This method increases the sensitivity to group differences compared to the standard linear approach, at the cost of being unable to separate linear and nonlinear effects.

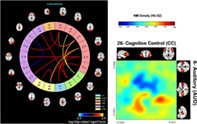

## 1 INTRODUCTION

During the past few decades, functional magnetic resonance imaging (fMRI) has become one of the most widely approaches for understanding brain function. In this area, functional network connectivity (FNC) has been widely used to analyze the relationship among distinct brain's regions (Allen et al., 2011; Bastos & Schoffelen, 2016; Friston, 2011; Sala‐Llonch et al., 2015; van den Heuvel & Hulshoff Pol, 2010). Most functional connectivity studies, concentrate on linear relationships between time courses. Studies on network models for fMRI data suggest linear correlation as a successful statistical tool to identify the relation between fMRI time courses (Smith et al., 2011), which is also easy to calculate and interpret for positive and negative correlations in the field (e.g., the default mode network tends to be anticorrelated to other networks).

However, some research has suggested that some of the brain activity exhibits nonlinear dynamic behavior (Lahaye et al., 2003; Stam, 2005; Su et al., 2013; Wismüller et al., 2014). Other studies discuss the nonlinear effects of hemodynamic responses in fMRI data (Deneux & Faugeras, 2006; Miller et al., 2001; Obata et al., 2004), which, importantly, can also vary with time (and location) and changes from subject to subject (de Zwart et al., 2009). Considering even just these few examples of nonlinear effects, it is likely, even expected, that distinct brain areas might be nonlinearly related in a way that would be missed by conventional linear analysis.

In the current study, we were interested in evaluating the degree to which explicitly nonlinear relationships (i.e., after removing the linear relationships) exist among brain regions in a functional connectivity context and identifying significant dependencies. To our knowledge, there has been little work studying explicitly nonlinear relationships in functional connectivity.

Despite being widely used in the field, the linear correlation coefficient measures only linear relationships and ignores nonlinear contributions. Other higher‐order statistical tools that can assess nonlinearity are not quite sensitive to the fMRI time course relationship (Smith et al., 2011). As a result, we proposed a new statistical tool to measure explicitly nonlinear dependencies. We focused on a normalized version of mutual information (MI), which is an information theoretic approach that has the advantage of being capable of measuring both linear and nonlinear dependencies. Early work evaluated MI to capture more general relationships (V. Calhoun et al., 2003). More recently, alternative metrics for functional connectivity, including MI, have been explored (Mohanty et al., 2020; Sundaram et al., 2020; Tedeschi et al., 2005; Tsai et al., 1999; Wang et al., 2015; Zhang et al., 2018). However, to our knowledge, we are the first group to assess the explicitly nonlinear relationships among brain networks to evaluate their unique aspects relative to the linear relationships.

In summary, the contributions of this article are as follows. We developed an approach that explores the nonlinear dependencies among functional brain networks. Our method calculates the nonlinear dependency by employing the mutual information among the residual dependence after removing the linear relationship using a regression scheme. To assess whether the nonlinear relationships were potentially meaningful, we first focus on whether the resulting FNC matrices exhibit modular relationships consistent with functional integration. Second, we evaluated whether the nonlinear FNC shows meaningful group differences in a dataset consisting of resting fMRI data collected from schizophrenia patients and healthy controls. Finally, we proposed a statistical method that provides an option to preserve the linear interpretation while also accounting for additional nonlinear dependency.

## 2 MATERIALS AND METHODS

### 2.1 Participants and preprocessing

In this work, we use the fBIRN dataset, which has been analyzed previously (Damaraju et al., 2014). The final curated dataset consisted of 163 healthy participants (mean age 36.9, 117 males; 46 females) and 151 age‐ and gender‐matched patients with schizophrenia (mean age 37.8; 114 males, 37 females). Eyes‐closed resting‐state fMRI data were collected at seven sites across the United States (Keator et al., 2016). Informed consent was obtained from all subjects before scanning in accordance with the internal review boards of corresponding institutions. Imaging data of one site were captured on the General Electric Discovery MR750 scanner, and the rest of the six sites were collected on Siemens Tim Trio System. Resting‐state fMRI scans were acquired using a standard gradient‐echo echo‐planar imaging paradigm: FOV of 220 × 220 mm (64 × 64 matrices), TR = 2 s, TE = 30 ms, FA = 770, 162 volumes, 32 sequential ascending axial slices of 4 mm thickness and 1 mm skip.

Data were preprocessed by using several toolboxes such as AFNI, SPM, and GIFT. Rigid body motion correction using the INRIAlign (Friston, 2011) toolbox in SPM was applied to correct for head motion. To remove the outliers, the AFNI3s 3dDespike algorithm was performed. Then fMRI data were resampled to 3 mm^3^ isotropic voxels. Then data were smoothed to 6 mm full width at half maximum (FWHM) using AFNI3s BlurToFWHM algorithm and each voxel time course was variance normalized. Subjects with larger movement were excluded from the analysis to mitigate motion effects during the curation process. For more details, refer to the study by Damaraju et al. (2014)).

### 2.2 Postprocessing

The GIFT (http://trendscenter.org/software/gift) implementation of group‐level spatial ICA was used to estimate 100 functional networks as ICA components. A subject‐specific data reduction step was first used to reduce 162 time point data into 100 directions of maximal variability using principal component analysis. Next, the infomax approach (Bell & Sejnowski, 1995) was used to estimate 100 maximally independent components from the group PCA reduced matrix. The ICA algorithm was repeated multiple times for stability of estimation, and the most central run was selected as representative (Du et al., 2014). Finally, aggregated spatial maps were estimated as the modes of component clusters. Subject specific spatial maps (SMs) and time courses (TCs) were obtained using the spatiotemporal regression back reconstruction approach (Calhoun et al., 2001; Erhardt et al., 2011) implemented in the GIFT software.

To label the components, regions of peak activation clusters for each specific spatial map were obtained. After ICA processing, to acquire areas of peak activation clusters, one sample *t*‐test maps are taken for each SM across all subjects and then thresholded; also, mean power spectra of the corresponding TCs were computed. The set of components as intrinsic connectivity networks (ICNs) was identified if their peak activation clusters fell within gray matter and showed less overlap with known vascular, susceptibility, ventricular, and edge regions corresponding to head motion. This resulted in 47 ICNs out of the 100 independent components. Running over 20 times ICASSO, the cluster stability/quality index for all except one ICNs was very high. After TCs were detrended and orthogonalized by considering estimated subject motion parameters, spikes were detected by AFNI3s 3dDespike algorithm and replaced by third‐order spline fit values. For more detail see Allen et al. (2012) and Damaraju et al. (2014). After processing, the fBIRN dataset resulted in a matrix of 159 time points × 47 components × 314 subjects, including 163 Control and 151 SZ subjects.

### 2.3 Mutual information approach

While linear correlation is the most widely used measure to describe dependence, it can completely miss nonlinear dependencies. An example to illustrate this shortfall is Anscombe's Quartet (Anscombe, 1973), which shows that four plots of various nonrandom data points have the same correlation coefficient despite their wildly different dependence structure. To measure the explicitly nonlinear relation between a pair of TCs, the approach applied in this research was to remove the linear correlation and calculate the residual dependence.

The Pearson product moment correlation coefficient, ρ, of time courses x and y is

$$\rho =\frac{\mathrm{Cov}\;\left(x,y\;\right)}{S_{x}S_{y}},$$

where $S_{x}$ and $S_{y}$ are, respectively, the sample standard deviations, and $\mathrm{Cov}\;\left(x,y\;\right)$ is the sample covariance between x and y.

The correlation coefficient mainly measures the linear dependence between two distributions. However, nonlinear dependence is not captured in the value of the correlation coefficient. Recent statistical approaches have been proposed to measure the correlation without underestimating the nonlinear dependency. One of these methods, mutual information (MI), measures both linear and nonlinear dependencies. The formula determines the value of MI is

$$\mathrm{MI}\left(x,y\right)=H\left(x\right)+H\left(y\;\right)-H\left(x,y\right).$$

where $H\left(x\right)$ and $H\left(y\right)$ are marginal entropies and $H\left(x,y\right)$ is the joint entropy. However, MI units are not standardized, making it hard to compare across subjects and datasets. Some of normalizing factors for NMI have been discussed by Kvalseth (2017) and included multiple options such as (1) min($H\left(x\right)$, $H\left(y\right)$), (2) $H\left(x\right)+H\left(y\;\right)$, and (3) max($H\left(x\right)$, $H\left(y\right)$). In this work, we used the latter as (Horibe, 1985) proved that it is a (normalized) similarity metric. The normalized MI (NMI) formula is

$$\mathrm{NMI}\left(x,y\right)=\frac{H\left(x\right)+H\left(y\;\right)-H\left(x,y\right)}{\max\left[H\left(x\right),H\left(y\;\right)\right]}.$$

In this work, our goal was to calculate *only* the nonlinear component of dependence. To do so, we measure the data's mutual information dependencies after removing the linear dependency. For a given time courses x and y, fitting a linear model $\bar{y}=\mathrm{\alpha x}+\beta$ gives the linear correlation between x and y. Here, $\bar{y}$ is the best linear estimation of y when x is given, the slope is denoted by α, and β is the *y*‐intercept. Next, we cancel the linear effect by calculating $z=y-\bar{y}$. The nonlinear dependency of x and z is the same as x and y. Next, we can use $\mathrm{NMI}\left(x,z\right)$ to evaluate the nonlinear dependency of x and y. To assure symmetricity, that is, $\mathrm{NMI}\left(x,y\right)=\mathrm{NMI}\left(y,x\right)$, we took the average of the results when switching x and y.

### 2.4 Simulated experiment

**FIGURE 1:**
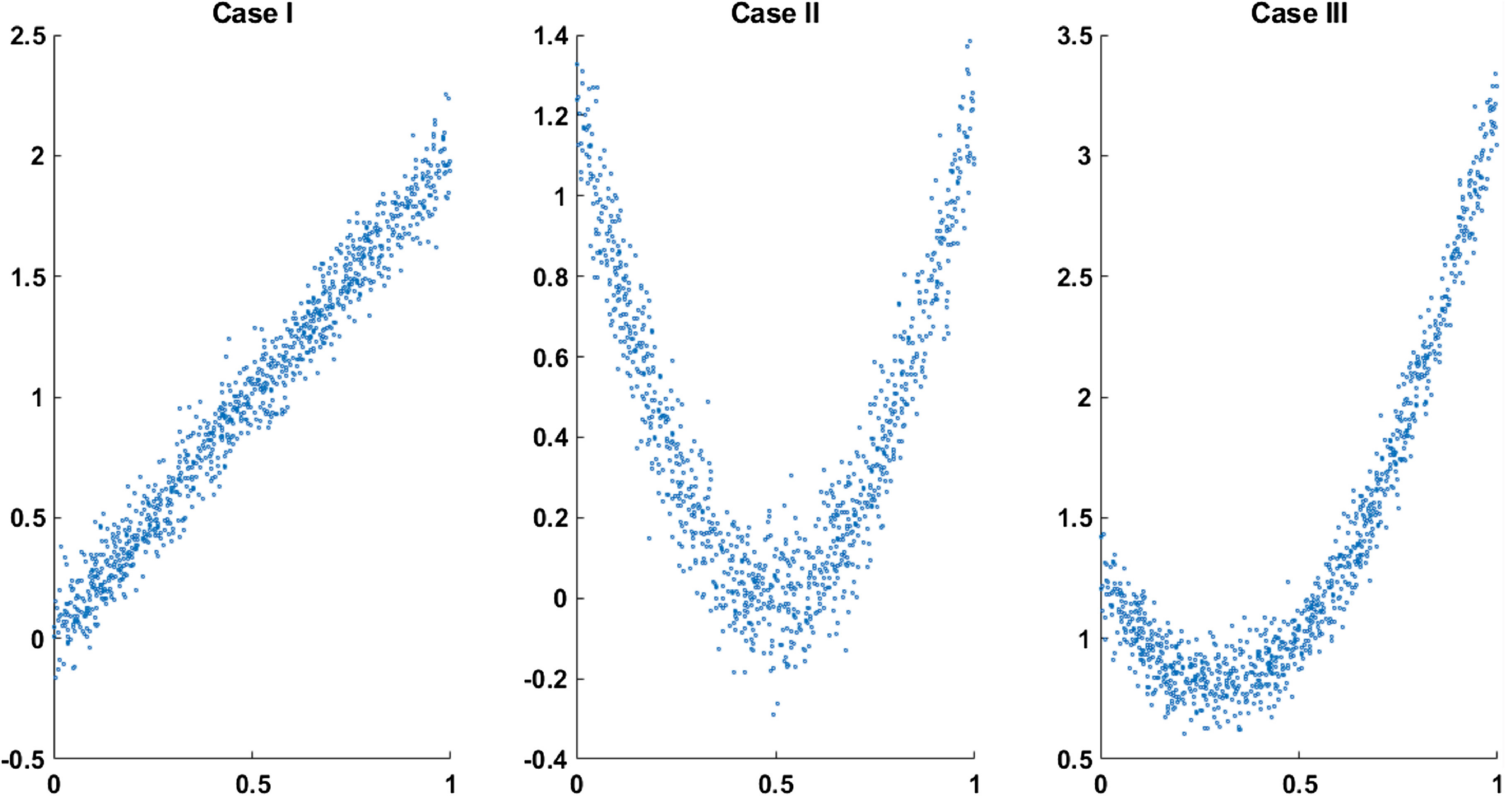
Three simulation cases for linear and nonlinear relationships between two vectors. Vector x has its components randomly derived from a uniform distribution on [0 1]. From left to right, we have Case I, Case II, and Case III such that in Case I, $\;y_{1}=2x+\varepsilon$ (linear relationship between x and $\;y_{1}$). In Case II, we have $y_{2}=5{\left(x-0.5\right)}^{2}+\varepsilon$ (nonlinear relationship between x and $\;y_{2}$) and for Case III, $y_{3}=5{\left(x-0.5\right)}^{2}+2x+\varepsilon$ (combination of linear and nonlinear relationships between x and $\;y_{3}$). Noise ε is a Gaussian distribution with a mean of zero

We applied the proposed method to simulated data to illustrate their use. In this experiment, we started with a vector say x of size 1000×1 where its components are from a random uniform distribution on [0 1]. Next, we formed three vectors $y_{1},y_{2},\text{and}\;y_{3}$, such that each one has a particular relationship with x. Three different types of relationships are as follows: Case I, vector $y_{1}$ has a purely linear relationship with x. Case II, we defined $y_{2}$ to have a quadratic relationship and no linear correlation with x. That is x and $y_{2}$ are only nonlinearly related. Case III, vector $y_{3}$ has a combination of linear and nonlinear dependencies with x. We also added zero‐mean Gaussian noise to $y_{1},y_{2},\text{and}\;y_{3}\;$(Figure 1).

We measured the relationship of $\left(x,y_{1}\right)$, $\left(x,y_{2}\right)$, and $\left(x,y_{3}\right)\;$ using both Pearson correlation and normalized mutual information approaches. Pearson correlation takes value from −1 to 1. Briefly, −1 refers to a perfectly linear negative correlation, and 1 shows a perfectly linear positive correlation. The normalized mutual information we use in this work is in the range of [0,1]. The NMI = 0 indicates no dependency, and as two distributions increase their dependence, the NMI value rises to a maximum of 1. Before computing correlation and NMI, we implemented the procedure explained earlier to remove the linear correlation from $y_{1},y_{2},\text{and}\;y_{3}$. Next, we calculated the Pearson correlation and normalized mutual information for each pair, as shown in Table 1.

**TABLE 1:** Simulation of three cases, including Case I: Linear correlation, Case II: Nonlinear relation and, Case III: Linear and nonlinear relationship between two vectors

	Corr_1_ $\left(x,\ldots \right)$	Corr_2_ $\left(x,\ldots \right)$	NMI_1_ $\left(x,\ldots \right)$	NMI_2_ $\left(x,\ldots \right)$
$\text{Case}\;I:y_{1}=2x+\varepsilon$	0.9847	1.64 × 10^−15^ (i.e., 0)	0.3809	0.0161
$\text{Case}\;\mathrm{II}:y_{2}=5{\left(x-0.5\right)}^{2}+\varepsilon$	−0.0271	−3.91 × 10^−17^ (i.e., 0)	0.2585	0.2582
$\text{Case}\;\mathrm{III}:y_{3}=5{\left(x-0.5\right)}^{2}+2x+\varepsilon$	0.8276	−1.61 × 10^−15^ (i.e., 0)	0.3139	0.2630

*Note*: The contribution of two vectors in each case was measured by Pearson correlation (Corr) and normalized mutual information (NMI). In this table, Corr_1_ and NMI_1_ show the correlation between the original data, and Corr_2_ and NMI_2_ show the correlation after removing the linear relationship. As expected, the correlation is effectively zero after the removal of linear effects. Results show that correlation completely misses the residual nonlinear dependencies, and that the NMI approach is able to effectively capture the nonlinear relationships when they exist.

### 2.5 Quantifying nonlinear connectivity in fMRI data

**FIGURE 2:**
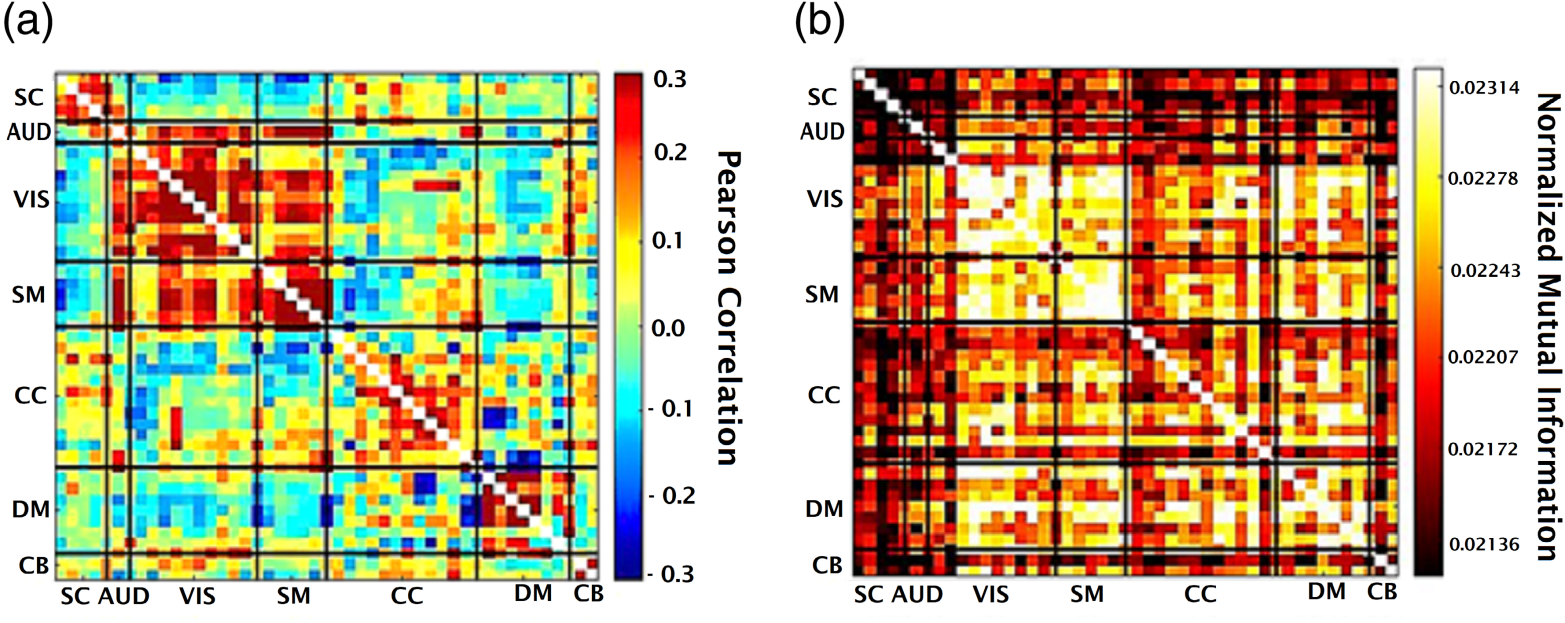
(a) Mean (linear) functional network connectivity (FNC) over 314 subjects. This range is [−0.3, 0.3]. (b) Mean mutual information (MI) after removing the linear correlation over 314 subjects, the range is [0.0203, 0.0242]. Note that both linear and nonlinear effects are modularized, but in different ways suggesting they are providing complementary information.

For each subject, there are 47 ICA time courses of length 159. For each pair of time courses x and y, we compute the traditional FNC (i.e., the linear correlation between all pairs x and y). Next, the mean FNC matrix is calculated overall 314 subjects (Figure 2a).

We then fit a linear model to estimate the linear correlation between x and y. After this, we remove the linear effect to study the remaining dependencies by updating y as $y=y-\bar{y}$. Next, we calculate the residual dependencies among functional network components in fMRI data via NMI. This produces a matrix of 47 by 47 for each subject in which the value in $\left(x,y\right)$ entry shows the nonlinear dependencies calculated by the NMI method for x and y. After that, we computed the average overall subjects (Figure 2b). Then, to evaluate whether the NMI showed significant variation across the brain, we performed a *t*‐test comparing the mean of each cell to that of the minimum to identify cells where the average in given cell is significantly greater than the minimum average cell. In addition, we used the random matrix analysis method (Vergara et al., 2018) to examine the modularity of the resulting explicitly nonlinear dependency matrix.

**FIGURE 3:**
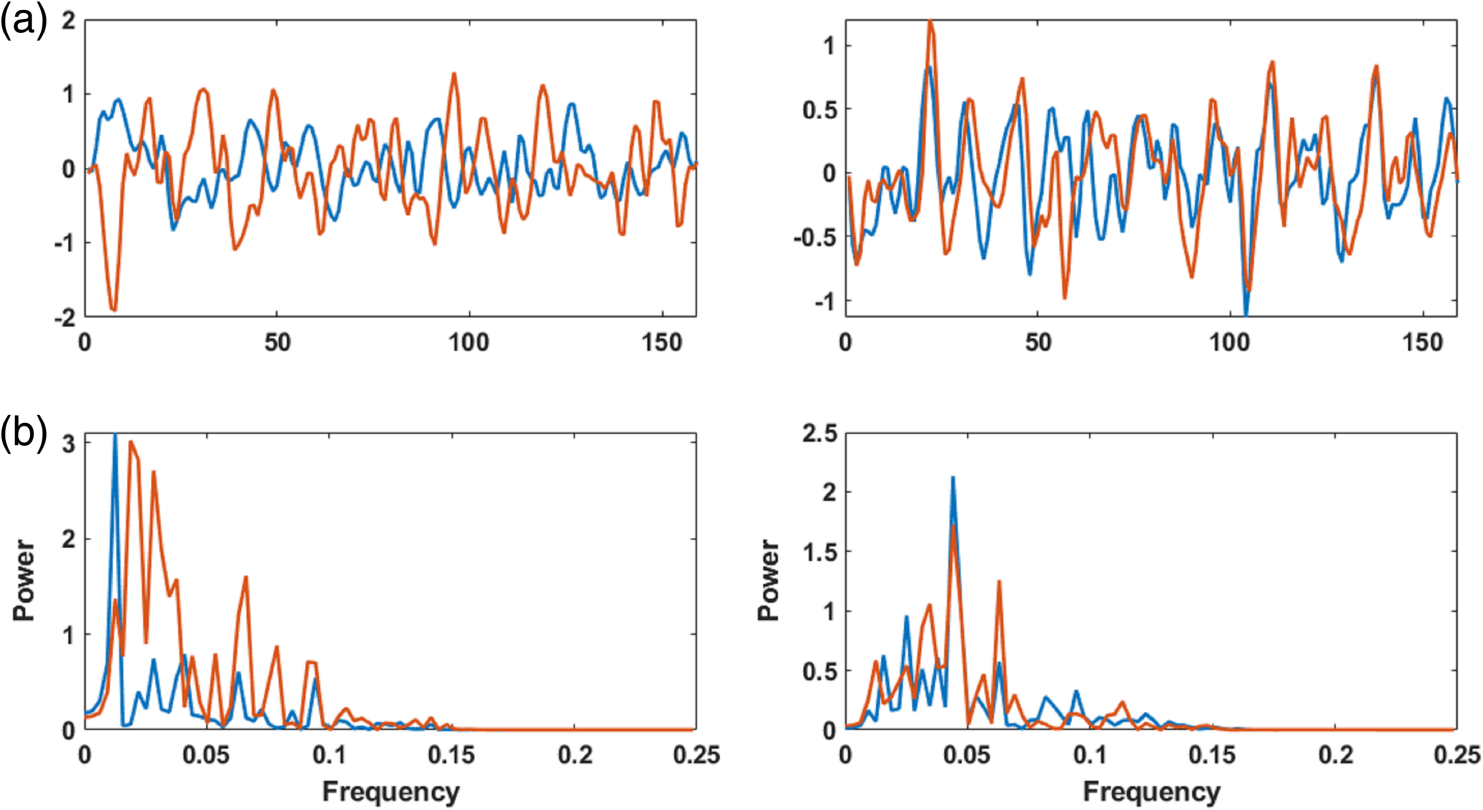
Linear and explicitly nonlinear relationship of two pairs of ICN components. The plots on the left are related to pair (23, 38) for one subject, and the plots on the right belong to the same subject for pair (2, 3). In panel (a), the nonlinear pair moves from a low to high coupling, where the high coupling links a higher frequency to a lower frequency pattern (right side of the figure on the left). Panel (b) displays the frequency spectrum. It shows when the two‐time courses exhibit dependencies but have different frequency profiles; linear correlations are not well capturing the relationships. This highlights one interesting aspect but note it may not be generally the case as these patterns vary across pairs and subjects.

Based on the result of linear FNC and explicitly nonlinear FNC in Figure 2, we select two extreme cells, pair (component #23 and component #38), which shows low linear correlation and high explicitly nonlinear dependence and pair (component #2 and component #3), which shows high linear correlation and low explicitly nonlinear dependency. For each pair, the TCs and their frequency spectrum are plotted in Figure 3.

We also compare the nonlinear dependencies between schizophrenia patients and controls. The linear effect is canceled within each group, and the average NMI is calculated over all subjects. Then, we implemented a *t*‐test to identify significant group differences. For false discovery rate (FDR) correction, all *p* values were adjusted by the Benjamini–Hochberg correction method and thresholded at a corrected *p* < .05.

### 2.6 Boosted approach

While we emphasize the unique information contained in the nonlinearities, future studies may wish to leverage both linear and nonlinear information. Pearson correlation is widely used in functional connectivity studies because of the simple calculation and being more sensitive to capture the dominant linear dependencies among the fMRI time courses. In addition, the sign of linear correlation reveals meaningful information about brain function. We propose a method that provides an option to preserve the linear interpretation while also accounting for additional nonlinear dependency. This *boosted* approach is a combination of Pearson correlation and modified mutual information for quantifying nonlinear dependencies as described in Section 2.3. We define the boosted method as

$$\begin{array}{l} \text{Pearson correlation}+\mathrm{sign}\text{(Pearson correlation)}\\ \;\times \text{Normalized Mutual information.} \end{array}$$

With this technique, we use the nonlinear information to boost the linear effects in the direction of the Pearson correlation, reflecting a stronger dependency. Thus, both linear and nonlinear relationships are considered, and the direction (which is not well defined in the nonlinear case) of the linear effect is preserved. The nonlinear and boosted methods that we propose allow for multiple uses. One can focus on the nonlinear effects only, which may be interesting in and of themselves, as we show in this article. Second, one can focus on capturing both linear and nonlinear effects in a “boosted” approach. This appears to increase sensitivity to group differences beyond the standard linear analysis, though it does not allow for linear and nonlinear effects separately. Note that NMI captures both linear and nonlinear effects; still, NMI shows somewhat reduced sensitivity to the linear effects. Also, NMI does not capture the directional relationships as NMI is always positive. The boosted approach retains the sensitivity and directionality of the linear relationships, which also provides some sensitivity to the nonlinear relationships.

We assessed the linear correlation (Pearson correlation), nonlinear dependencies (modified mutual information introduced in Section 2.3), and both linear and nonlinear dependencies (boosted) in schizophrenia patients and healthy controls components. Then separately for each method, a *t*‐test was applied, and *p* values were adjusted by the Benjamini–Hochberg correction method and threshold at a corrected *p* < .05.

### 2.7 Joint distributions

To visualize the identified nonlinear relationships further, we selected the five component pairs with the most significant *p* values in the *t*‐test for group differences in the nonlinear dependence for HC‐SZ. Then we constructed the difference in the joint distributions for each pair of time courses, comparing patients and controls.

## 3 RESULTS

### 3.1 Simulated experiment

Three types of dependencies: linear, nonlinear, and a combination of linear and nonlinear, are examined. The Pearson correlation and mutual information before and after removing the linear dependency for each case are measured and reported in Table 1.

The range of Pearson correlation is −1 to +1, and the range of normalized mutual information for independent distributions is 0, and the perfect dependency is 1. In Case I, where the two distributions have only a linear correlation, the Pearson correlation is close to one before removing the linear effect. After removing, both Pearson and normalized mutual information are close to zero. In Case II, where the two distributions have a quadratic relationship, the Pearson correlation shows a low but non‐zero correlation. In comparison, the normalized mutual information calculation shows a considerable correlation between the two distributions. After removing the linear effects, the Person correlation is effectively zero while the mutual information is approximately the same before and after removing the linear effect. In Case III, where there is both linear and nonlinear relationships between two distributions, the Pearson correlation is significant before removing the linear effect. It vanishes after canceling the linear correlation, while the normalized mutual information only slightly decreases after removing the linear correlation. This briefly demonstrates that Pearson correlation does not capture purely nonlinear dependencies, while mutual information considers both linear and nonlinear dependencies. Similarly, if we remove the liner effect, the correlation will go to zero, whereas the mutual information will capture the true residual nonlinear dependencies between two distributions.

### 3.2 On fMRI data

We measured the linear correlation among 47 ICN's time courses estimated from the resting fMRI data—the result in Figure 2. Figure 2a is the average FNC across 314 subjects.

The average of nonlinear dependencies across all subjects is calculated using our proposed NMI method for the same components (Figure 2b). The assessment of randomness confirms that explicitly nonlinear FNC indicates highly significant modularity relative to a random matrix. Then, a *t*‐test for one sample is applied to identify pairs with a significant difference in average value from the minimum average. After FDR thresholding (*p* < .05), most pairs are detected to be significantly larger than the minimum mean. This shows us the degree to which NMI differences are significantly different across the FNC matrix and demonstrates modular nonlinear dependencies between visual (VIS) and somatomotor (SM) components with other components. Interestingly, a low nonlinear dependency rate is observed despite a high linear correlation between subcortical (SC) and auditory (AUD).

For one subject, the time courses of pair (23,38) and (2,3) are plotted (Figure 3a), and their spectrum using the amplitude of the fast Fourier transform (FFT) are calculated (Figure 3b). Across all subjects, pair (23,38) shows relatively high explicitly nonlinear dependencies (0.0236) and low linear correlation (−0.0127). Pair (2,3) shows a relatively high linear correlation (0.2194) on average overall subjects but low explicitly nonlinear dependency (0.0208).

**FIGURE 4:**
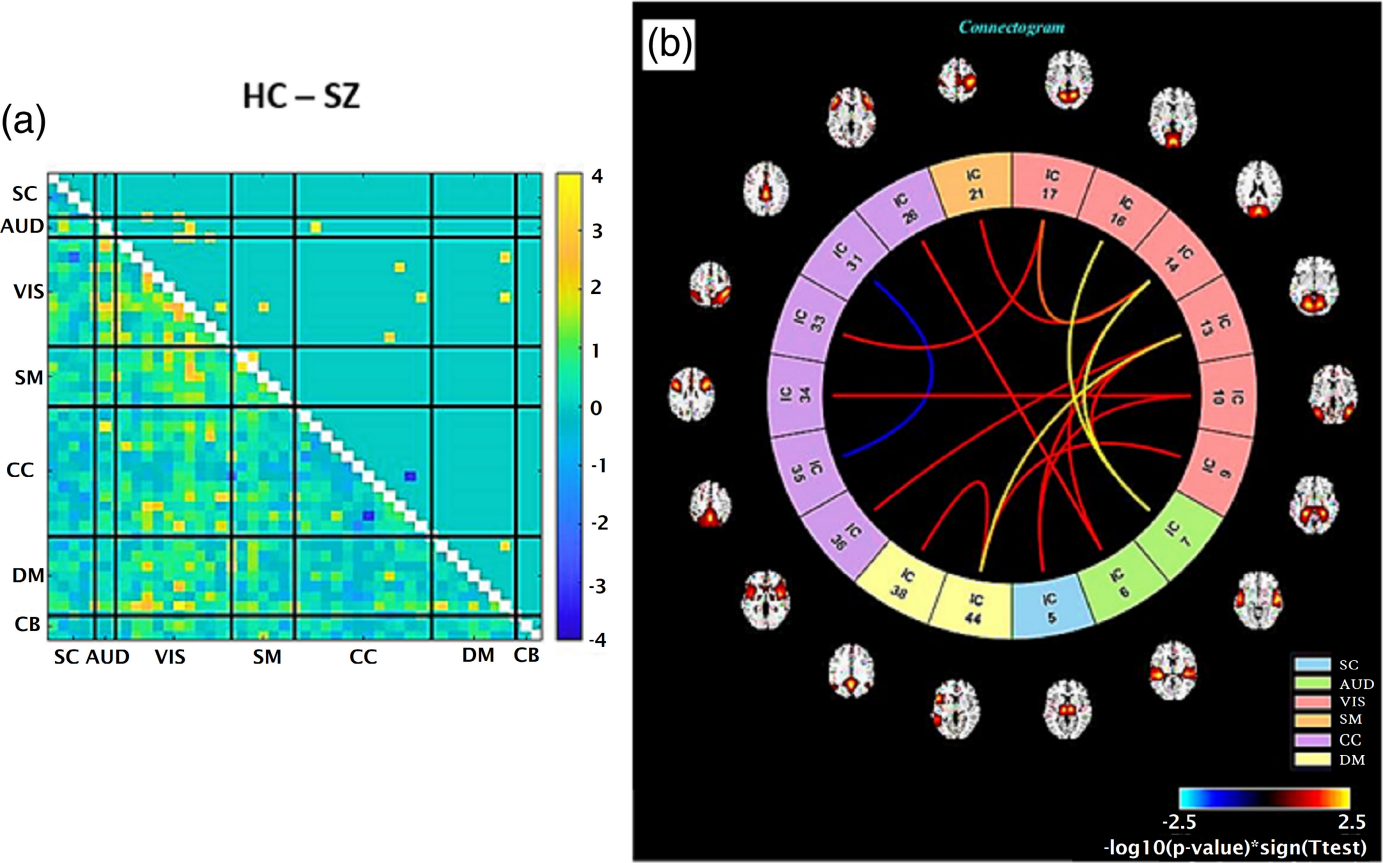
(a) Upper triangle: The group difference (HC‐SZ) in NMI after removing the linear correlation. The *p* values are adjusted by FDR and threshold (*p* < .05). Values are plotted as −log10(*p* value) × sign(*t*‐statistics). Lower triangle: Identical to the upper tringle except that *p* values are not threshold, also the values are multiplied by the initial *p* value before FDR. (b) Connectogram that shows components with significantly different nonlinear relationships in HC‐SZ are connected. In all but one case, results show significantly more nonlinearity in the controls, mostly linked to the visual domain.

Next, the variation in nonlinear dependencies between healthy controls and schizophrenia patients is evaluated using our NMI method and compared using a two‐sample T‐test. In Figure 4a, the lower triangle shows $-\log10\left(p\right)\times \mathrm{sign}\left(T\right)$ before threshold multiply by the initial *p* value before FDR. The upper triangle is the same as the lower one after thresholding. Entries shown in a different color identify pairs with significant differences in nonlinear dependency between groups. This result shows significant differences in nonlinear dependencies between visual (VIS) components to other components such as auditory (AUD), visual (VIS), somatomotor (SM), cognitive control (CC), and default‐mode (DM) in schizophrenia (SZ) patients relative to healthy controls (HC). Panel b, the connectogram, is another representation of the t‐test result. The spatial ICNs connected with a line if the difference in their nonlinear dependencies between HC and SZ is significant (with yellow for HC > SZ and blue for SZ > HC).

### 3.3 Boosted approach

Dependencies among components in healthy controls and schizophrenia patients are assessed with three methods: (1) Pearson correlation, in which the emphasis is on only linear correlation, (2) modified mutual information, as described in Section 2.3, quantifies only nonlinear dependencies, and (3) the boosted approach is explained in Section 2.6, is boosting the linear correlation by capturing nonlinear dependencies.

Next, in each method, the *t*‐test is applied to compare the differences between two groups. Adjusted *p* value by FDR is thresholded (*p* < .05). The number of pairs with significant differences for the Pearson correlation method is 530, the modified mutual information method is 17, and the boosted method is 537. There are five pairs for which their linear correlation is not significantly different, but their nonlinear dependencies are significant. Note that linear and explicitly nonlinear analyses are complementary to one another. The explicitly nonlinear NMI approach gives 17 significant pairs regardless of whether their linear dependency is significant or not. That is, this information and differences in nonlinear changes are completely ignored by the linear correlation approach and importantly, the brain wide pattern of nonlinear group differences is highly modular and structured. The boosted method provides an option to capture both the linear and nonlinear components of dependencies. Results also show the boosted approach identifies relationship that is not captured by either the linear or full NMI approaches.

### 3.4 Joint distributions

**FIGURE 5:**
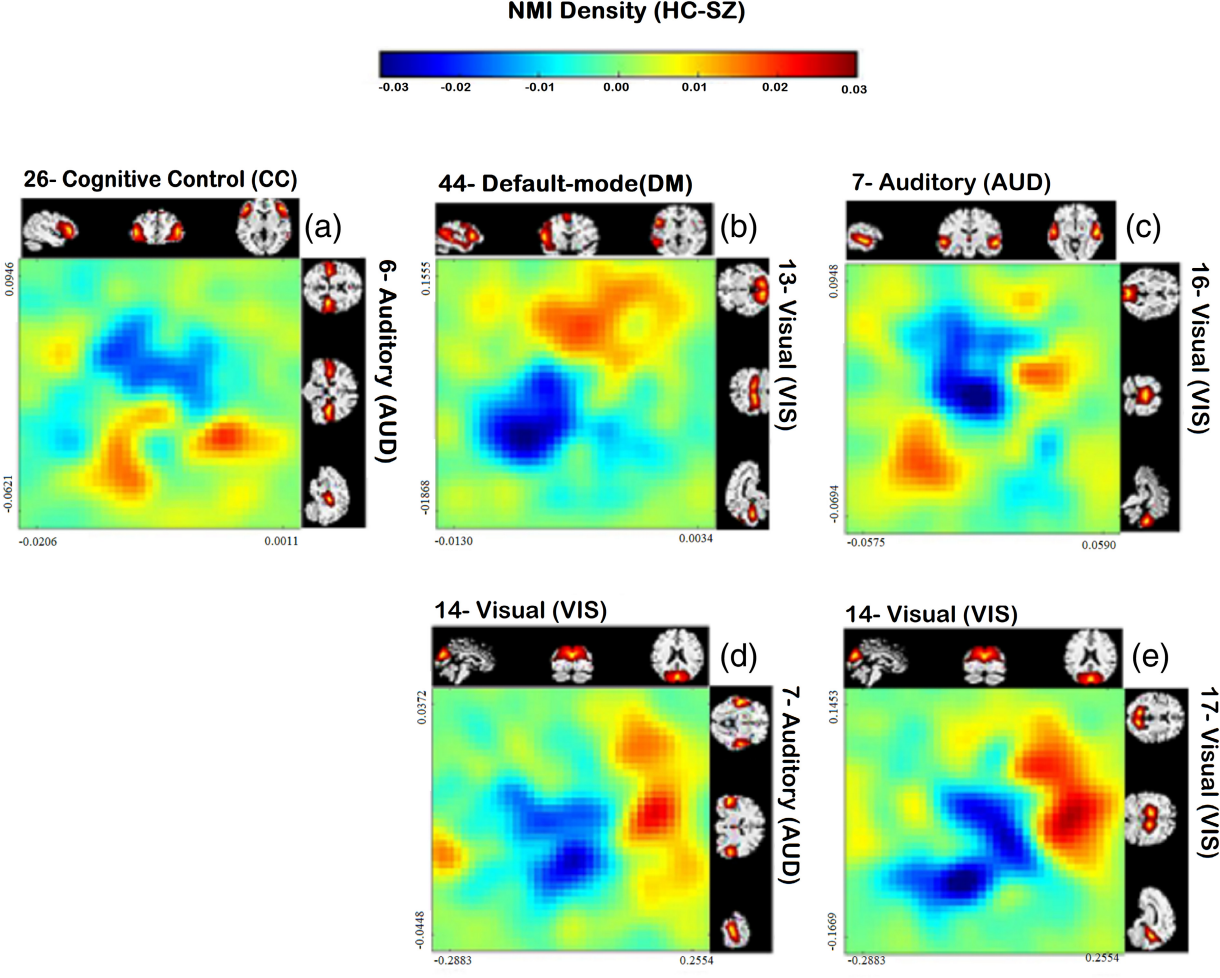
Difference between the HC and SZ joint distributions for the five pairs showing the largest group differences in the nonlinear dependencies of pairs (26, 6), (44, 13), (7, 16), (14, 7), (14, 17). Values in each distribution increases from left to right and down to up. The 26th component belongs to cognitive control (CC). The sixth and seventh components are in auditory (AUD). The 44th component is in default mode (DM). All 13th, 14th, 16th^,^ and 17th components are in the visual (VIS) domain. We observe some interesting differences in the joint distributions, with patients generally showing differentially higher activity in one network and lower activity in the paired network.

We were also interested in visualizing the relationship among time course pairs, which exhibited nonlinear differences between patients and controls, Figure 5 demonstrates the differences in joint distribution between healthy controls and schizophrenia patients for the five pairs showing the largest group differences. Values increases from left to right and down to up. Panel a shows the difference in the joint distribution of the 26th and 6th components. The 26th component belongs to cognitive control (CC) 6th component is in the auditory (AUD) domain. Panel b is the joint distribution difference of the 44th and 13th components. The 44th component is in default mode (DM), and the 13th component is in the visual (VIS) domain. Panel c represents the joint distribution difference of the 7th and 16th components. The 7th component is auditory (AUD), and the 16th component is visual (VIS). Panel d illustrates the difference in the joint distribution of the 14th and 7th components, and Panel e exhibits the joint distribution of the 14th and 17th components. Both 14th and 17th components belong to the visual (VIS) domain. Panels b, d, and e share some similarities, including a negative relationship between the two components.

In Figure 5a, we can observe controls spend more time in the low level of auditory component #6 relative to SZ regardless of the values of the 26th component. From Figure 5b, healthy controls are considerably more active in both the default mode network (#44) and a visual component (#13) than in SZ. In Panel c, we notice healthy controls show less activity in both auditory (#7) and visual (#16) components compared to SZ. From Figure 5d,e, it can be interpreted those healthy controls show a higher level of activation in visual (#14) components relative to a more posterior visual (#17) and an auditory (#7) component than do the patients.

For these five pairs, nonlinear FNC shows hyper‐connectivity compared to SZ, that is the nonlinear part of two distributions has more dependency in HC. However, the plot of group differences in the joint distribution can illustrate how two pairs that show high dependence in HC can be differently distributed in SZ. Panels b,d and e shows healthy controls are mostly in the higher levels of activation (red color is dragged to the upper corner and blue to the down left). In contrast, Panel c shows healthy controls tend to show lower activation levels (red is dragged to the lower left and blue to the upper right).

## 4 DISCUSSION

In this preliminary work, we highlight the benefit of studying nonlinearities in functional connectivity. Previous functional connectivity studies are based on correlation coefficients that assess the linear correlation only, and as a result, they miss the nonlinear contributions. We establish an approach to evaluate the explicitly nonlinear dependencies between distinct brain regions by first removing the linear dependencies. We first demonstrated our approach works as expected on simulated data (Figure 1). Following the nonlinear dependencies among 47 time courses on 314 subjects are assessed (Figure 2). A similar approach was applied to estimate how differently in average distinct regions of a schizophrenia patient's brain contributes nonlinearly to the context of functional connectivity (Figure 4). Also, the joint distribution of five pairs with the largest group differences in the nonlinear dependencies in HC‐SZ is studied (Figure 5).

There are a number of possible causes of nonlinear dependencies, including (1) nonlinear hemodynamic effects. Studies on the relationship between neuronal activity, oxygen metabolism, and hemodynamic responses have shown the link between neuronal activity and hemodynamic response magnitude exhibits both linear and nonlinear effects in task data (Friston et al., 2000; Sheth et al., 2004; Wan et al., 2006). Nonlinearity can be induced via hemodynamic response changes with time. The hemodynamics can also vary between subjects and groups. Other results suggest a strongly nonlinear relationship between electrophysiological measures of neuronal activity and the hemodynamic response (Devor et al., 2003; Sheth et al., 2004). (2) Differences in blood flow, blood oxygenation, and blood volume both within subjects and between groups. Experiments indicate that acquired vascular space occupancy (VASO), arterial spin labeling (ASL) perfusion, and BOLD signals respond nonlinearly to stimulus duration (de Zwart et al., 2009; Gu et al., 2005). (3) Subject motion. Even minor head posture changes may result in considerable spatially complex field changes in the brain (Liu et al., 2018). While we cannot completely exclude motion, we carefully curated the data to focus on low motion subjects and in addition, there were no significant motion differences between the groups (Damaraju et al., 2014).

The different modularized patterns evident in linear and nonlinearly modularity suggest a complementarity of the nonlinear and linear relationships. It may be important to capitalize on these differences in future studies. Our results suggest an interesting variation among networks. For example, as shown in Figure 5b, significant nonlinear dependencies are observed between visual (VIS), somatomotor (SM) domains and within cognitive control (CC) and default‐mode (DM) domains. The auditory (AUD) network shows strong differences in linear dependencies (a), but not much nonlinear, whereas both visual and sensorimotor show strong within domain nonlinear dependencies (b). Also, a relatively low rate of nonlinear dependencies is observed between subcortical (SC) and auditory (AUD) with other components.

We also found significant differences in the nonlinear relationships among the patients and controls. Nonlinear FNC pairwise comparisons between SZ and HC are shown in Figure 5a. In most cases, the controls are showing higher nonlinear dependencies relative to patients, mostly linked to the visual domain. There are a significant differences in nonlinear relation within visual (VIS) components as well as between VIS components and to other components such as auditory (AUD), somatomotor (SM), cognitive control (CC), and default‐mode (DM) in SZ patient and HC. We observe that most of the patient/control differences involve visual and auditory components. This is intriguing given existing evidence suggesting some schizophrenia symptoms may be linked to the visual system (Gong et al., 2020; Johnston et al., 2005; Onitsuka et al., 2007). Having said that, visual symptoms such as visual hallucinations are rather uncommon in SZ and rarer than auditory and tactile abnormalities (van de Ven et al., 2017). In addition, some studies suggest inborn blindness may be shielded against the development of schizophrenia, characterized by inevitably noisy perceptual input that causes false inferences. These findings argue that when individuals cannot see from birth, they depend more on the other senses. Thus, the resulting model of the world is more resistant to false interpretations (Landgraf & Osterheider, 2013; Leivada & Boeckx, 2014; Morgan et al., 2018; Pollak & Corlett, 2019; Riscalla, 1980).

While the results presented show the potential utility of focusing on explicitly nonlinear dependencies in fMRI data, there is still much work to be done. Future work should focus on carefully evaluating the possible sources of the nonlinear relationships. Quantitative fMRI studies could be used to isolate nonlinearities in blood oxygenation, volume, and flow. In addition, high field layer‐specific fMRI studies could be used to evaluate nonlinearities in input vs. output layers. The contribution of various physiological variables (e.g., respiration, CO_2_, heart rate, and motion) could also be evaluated in future work.

In addition, our results provide evidence suggesting there are meaningful and significant nonlinear dependencies among fMRI time courses. We have shown evidence suggesting meaningful (modularized and group different) super‐linear effects in FNC, which primarily implicates the visual cortex as disrupted in schizophrenia. We present two approaches, focusing on the explicitly linear effects or a boosted method that captures both linear and nonlinear effects within one metric. Future work should study the information contained in the nonlinear relationships. It could be studied with faster acquisitions, linked to multimodal imaging such as concurrent EEG data, and replicate the results we show in this work.

## CONFLICT OF INTEREST

No competing financial interests exist.

## Supporting information


**Appendix S1**: Supporting Information

## Data Availability

Supplementary data to this article can be found online at http://dx.doi.org/10.1016/j.nicl.2014.07.003
